# Blood-Based Biomarkers Reflecting Protease 3 and MMP-12 Catalyzed Elastin Degradation as Potential Noninvasive Surrogate Markers of Endoscopic and Clinical Disease in Inflammatory Bowel Disease

**DOI:** 10.3390/jcm13010021

**Published:** 2023-12-19

**Authors:** Martin Pehrsson, Viktor Domislovic, Marta Sorokina Alexdottir, Marko Brinar, Morten Asser Karsdal, Ana Barisic, Zeljko Krznaric, Joachim Høg Mortensen

**Affiliations:** 1 Biomarkers and Research, Nordic Bioscience A/S, 2730 Herlev, Denmark; mara@nordicbio.com (M.S.A.); mk@nordicbio.com (M.A.K.); jhm@nordicbio.com (J.H.M.); 2 Department of Gastroenterology and Hepatology, University Hospital Center Zagreb, 10000 Zagreb, Croatia; viktor.domislovic@gmail.com (V.D.); brinarm@gmail.com (M.B.); zeljko.krznaric60@gmail.com (Z.K.); 3 Center for Clinical Nutrition, University Hospital Centre Zagreb, 10000 Zagreb, Croatia; dr.ana.barisic@gmail.com

**Keywords:** biomarker, IBD, elastin remodeling, Crohn’s disease, ulcerative colitis, neoepitope, blood-based biomarker, endoscopic disease, clinical disease

## Abstract

Chronic inflammation in inflammatory bowel disease (IBD) triggers significant extracellular matrix remodeling, including elastin remodeling, leading to severe clinical complications. Novel methods to assess intestinal tissue destruction may act as surrogate markers of endoscopic disease activity, relieving patients of invasive endoscopy. We explored the noninvasive blood-based biomarkers ELP-3 and ELM-12, measuring elastin degradation in IBD. In a study involving 104 Crohn’s disease (CD), 39 ulcerative colitis (UC), and 29 healthy donors, we assessed these biomarkers’ association with endoscopic and clinical disease activity using ELISA. Patients were evaluated based on the SES-CD and CDAI for CD patients and modified MES and partial Mayo for UC patients. ELP-3 and ELM-12 were elevated in patients with IBD. Discerning CD patients in endoscopic remission and mild from moderate to severe, ELP-3 provided an AUC of 0.69 and ELM-12 an AUC of 0.73. The ELP-3 biomarker was associated with UC patients and provided the highest diagnostic power of 0.87 for remission vs. active clinical disease. The data suggest an association of ELP-3 with active CD and ELM-12 with endoscopic remission in CD patients. Additionally, ELP-3 could identify UC patients with active clinical disease from patients in remission. The noninvasive biomarkers ELP-3 and ELM-12 could be potential surrogate biomarkers of elastin degradation and endoscopic and clinical disease markers.

## 1. Introduction

IBD encompasses Crohn’s disease (CD) and ulcerative colitis (UC), chronic inflammatory conditions affecting the gastrointestinal tract [1,2]. The disease is triggered by genetic factors and environmental influences, leading to persistent inflammation and tissue damage [3]. Tissue destruction driven by immune cells plays a crucial role in IBD, impacting the extracellular matrix (ECM) of the intestinal tissue [4,5]. Elastin, a significant ECM component of the intestinal tissue, provides elasticity and strength to the intestine [5]. Immune cell-driven protease activity, including matrix metalloproteins (MMPs), protease 3 (PR3), and neutrophil elastase, can cause elastin degradation, leading to complications like intestinal bleeding and fistula formation [6,7].

Current and emerging treatments aim to restore tissue homeostasis and integrity [8,9,10,11], primarily using endoscopy as an assessment tool. However, due to the invasiveness and discomfort of the procedure, routine endoscopies are not performed, requiring novel noninvasive surrogate markers of mucosal healing and tissue destruction.

Previous studies on ECM remodeling in IBD focused on collagen formation and degradation [12,13,14]. This study investigated elastin degradation catalyzed by proteases related to neutrophils and macrophages in CD and UC patients. Elastin is a vital element of elastic fibers, providing tissue elasticity and durability due to molecular crosslinks protecting against proteolysis [15]. However, over time, elastic fibers accumulate damage, becoming vulnerable to elastin-degrading proteases, such as MMPs and serine proteases [16]. In IBD, chronic inflammation and increased proteolytic activity may lead to elevated elastin degradation, perpetuating the disease and producing signaling fragments known as elastokines [16]. Proteomic analysis of UC tissue biopsies revealed reduced elastin expression and increased PR3 expression, an elastin-degrading neutrophil-derived protease [17,18]. Another relevant protease in IBD, MMP-12 from macrophages, exhibits elastolytic activity [16] and contributes to tissue destruction in CD [19]. 

**Figure 1:**
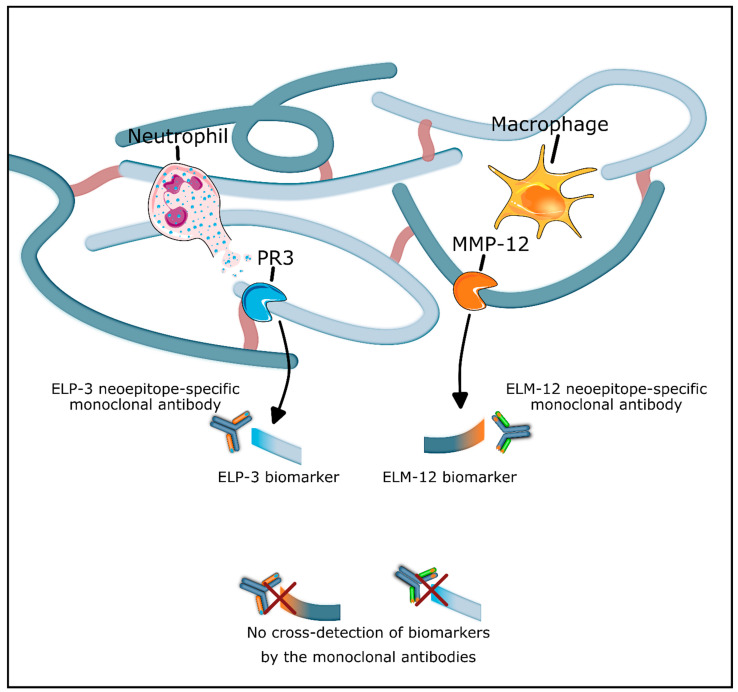
Generation and detection of the ELP-3 and ELM-12 biomarkers. The proteolytic activity of protease-3 (PR3) and matrix metalloproteinase (MMP)-12 from neutrophils and macrophages results in the generation of neoepitope elastin fragments (ELP-3 and ELM-12). The elastin fragments can be detected and quantified using neoepitope-specific monoclonal antibodies in an enzyme-linked immunosorbent assay. The monoclonal antibodies are highly specific for their respective neoepitope with no cross-detection.

Considering the significance of elastin in intestinal tissue and its susceptibility to proteolytic degradation, we sought to investigate two neoepitope-specific blood-based biomarkers in patients with IBD. The two biomarkers, ELP-3 and ELM-12, quantify PR3 and MMP-12-catalyzed elastin degradation, objectively measuring immune cell-mediated elastin remodeling (Figure 1). Additionally, we investigated whether the biomarkers can act as noninvasive surrogate markers of disease activity in IBD. Measuring these biomarkers allows objective assessment of protease-specific elastin degradation and elastolytic activity, linking the findings to disease activity.

## 2. Materials and Methods

### 2.1. Patient Information

In this cross-sectional study, we evaluated two biomarkers of elastin degradation in 143 IBD patients (CD = 104 and UC = 39) and 29 age- and gender-matched healthy donors (Valley Biomedical, Winchester, VA, USA). The healthy donor samples were collected at FDA-licensed commercial donor centers within the US and screened negative for serological disease state markers. Demographic information, disease history, and therapy details were collected from electronic medical records and patient questionnaires. Only adult patients with a confirmed IBD diagnosis were included, and anthropometric parameters were measured at enrollment. Patients with systemic infection, bowel infections, other inflammatory conditions, and extraintestinal inflammation were excluded. CD and UC were classified based on the Montreal Classification upon inclusion. The Zagreb University Hospital Center ethics committee approved the study, and all participants provided signed informed consent before enrollment. All methods were performed following relevant guidelines and regulations.

### 2.2. Endoscopic and Clinical Disease Definitions

We determined disease activity in CD and UC patients by analyzing blood samples and using specific scoring systems. For CD patients, we used the Simple Endoscopic Score for Crohn’s Disease (SES-CD) (*n* = 59) and the modified Mayo Endoscopic Score (mMES) (*n* = 32) for UC patients. The mMES assesses disease extension and severity of inflammation by scoring different colon segments and calculating the maximum extension of the disease [20]. Clinical disease activity was evaluated using the Crohn’s Disease Activity Index (CDAI) (*n* = 104) and partial Mayo score (pMayo) (*n* = 39). Patients were categorized based on the endoscopic and clinical disease scores as follows: SES-CD (remission 0–2, mild 3–6, moderate 7–15, severe >15), mMES (remission 0–2, mild 3–6, moderate 7–15, severe >15), CDAI (remission ≤150, mild 151–219, moderate 220–450, severe >450), and pMayo (remission <2, mild 2–4, moderate 5–7, severe >7).

### 2.3. Biomarker Analysis

Two biomarkers, ELP-3 and ELM-12, reflecting elastin degradation catalyzed by PR3 and MMP-12, were assessed in the patient’s blood samples. Blood was collected in the morning after an overnight fast. The biomarkers were measured in the serum using competitive enzyme-linked immunosorbent assays [21,22]. Then, 96-well plates pre-coated with streptavidin (Roche Diagnostics cat. no.11940279, Hvidovre, Denmark) were incubated with a biotinylated antigen corresponding to the neoepitope of ELP-3 or ELM-12 for 30 min at room temperature. Subsequently, serum samples and controls were incubated with a horseradish peroxidase-conjugated neoepitope-specific monoclonal antibody diluted in an incubation buffer containing 1% bovine serum albumin (cat.no. a-7906, ≥98 purity, Sigma Aldrich). After each incubation step, the wells were washed in washing buffer (25 mM TRIZMA, 50 mM NaCl, 0.036% Bronidox L5, and 0.1% Tween-20), and tetramethyl benzidine (TMB, Kem-EN-Tec cat. no. 438OH, Taastrup, Denmark) was added for 15 min at room temperature. Subsequently, 0.18M H_2_SO_4_ was added, and the optical density at 450 nm and 650 nm was measured using a SpectraMax M5 (Molecular Devices, San Jose, CA, USA). The biomarker concentration was determined by extrapolating the optical density to the generated standard curve using Softmax Pro 7 (Molecular Devices, San Jose, CA, USA).

### 2.4. Statistical Analysis

Patient characteristics were summarized using descriptive statistics. Categorical variables were presented as patient numbers and percentages, while continuous variables were expressed as medians with interquartile ranges (IQR). Biomarker levels were compared between CD, UC patients, and healthy donors and displayed as box plots with the median and 10th–90th percentile range. A correlation analysis was performed to determine the association between confounders (age, body mass index (BMI), gender, biological therapy status (yes/no), ileal resection (yes or no), smoking status (yes/no/ex)), and disease duration (years)) and the biomarkers (ELP-3 and ELM-12). Subsequently, ROC analysis was employed to assess the discriminative ability of ELP-3 and ELM-12 in distinguishing patients with CD or UC based on endoscopic and clinical scoring using the DeLong et al. methodology. Statistical analysis was carried out using MedCalc version 19.3 (MedCalc Software Ltd., Ostend, Belgium).

## 3. Results

### 3.1. Patient Demographics

The median age of CD and UC patients was 36 and 38 years, respectively, comparable to healthy donors (39.5 years) (*p* > 0.999). CD patients had a higher, albeit non-significant, percentage of males (63.5% vs. 59%) (*p* = 0.829), current smokers (22.1% vs. 12.8%) (*p* = 0.246), and those on biological therapy (59.6% vs. 56.3%) (*p* = 0.336) compared to UC patients. Most CD patients (65.0%) had ileocolonic (L3) disease, while 17.5% and 17.5% had ileal (L1) and colonic (L2) disease, respectively. Based on endoscopy, 39.5% of CD patients had luminal disease (B1), and 40.7% and 19.8% had strictures (B2) or penetrating disease (B3). About half (50.8%) of CD patients were in endoscopic remission, with 18.0% and 31.1% having mild or moderate to severe endoscopic disease according to their SES-CD scores. A total of 50.0% of CD patients underwent ileal resection.

In UC patients, 60.0% had pancolitis (E3), 10.0% had proctitis (E1), and 30.0% had left-sided colitis (E2). The proportion of UC patients with mild to severe endoscopic disease was higher than those in endoscopic remission (28.1% vs. 71.9% for mMES). Among CD patients, 77.9% were in remission based on the CDAI score for clinical activity, while 13.5% and 8.7% had mild and moderate to severe disease, respectively. For UC patients, the largest proportion was in clinical remission according to the pMayo score (53.8%), compared to 23.1% with mild and 23.1% with moderate to severe disease (Table 1). Patients with CD had a signficantly longer disease duration than patients with UC with a median of 9.0 years compared to 6.0 years (*p* = 0.014). No significant difference in CRP levels was observed (*p* = 0.884)

### 3.2. Elevation of Proteolytic Elastin Degradation in Patients with IBD

**Figure 2:**
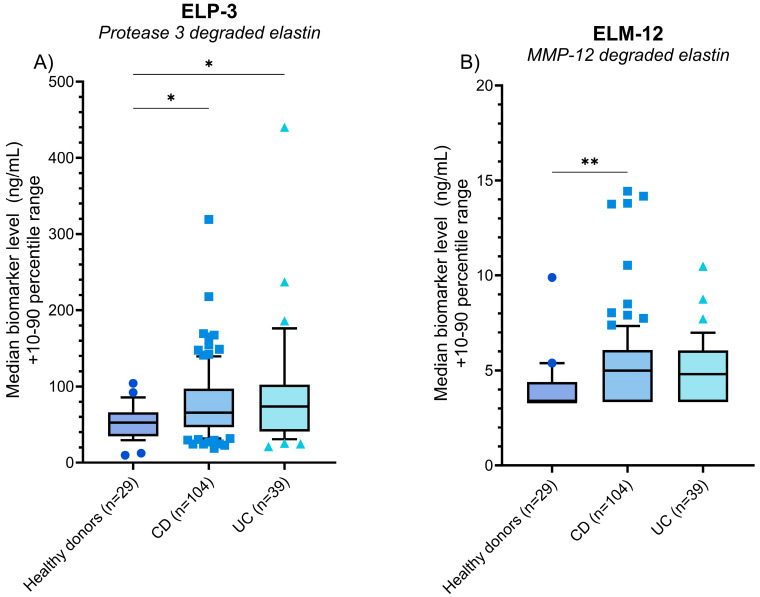
Biomarker levels of ELP-3 (**A**) and ELM-12 (**B**) quantified in the serum of healthy donors (*n* = 29), patients with CD (*n* = 104), and patients with UC (*n* = 39). The data are plotted as box plots showcasing the median and 10th–90th percentile biomarker levels, calculating the statistical difference between the healthy donors and patients with IBD using Kruskal–Wallis one-way ANOVA and applying Dunn’s test for multiple comparisons. The statistical significance between groups is presented as * *p* < 0.05 and ** *p* < 0.01.

We initially evaluated the differences in PR3 (ELP-3)- and MMP-12 (ELM-12)-catalyzed elastin degradation between patients with CD and UC and a group of age and gender-matched healthy donors (*n* = 29). Significantly higher levels of ELP-3 were detected in the serum of CD and UC patients compared to the healthy donors (*p* = 0.027 and *p* = 0.039) (Figure 2A). For the ELM-12 biomarker, CD patients demonstrated significantly increased biomarker levels compared to healthy donors (*p* = 0.001), with a numerical increase of ELM-12 in UC patients compared to healthy donors (median values (UC vs. healthy donors): 4.81 ng/mL vs. 3.34 ng/mL) (Figure 2B). 

### 3.3. Elastin Degradation Biomarkers Are Associated with Endoscopic and Clinical Disease in CD Patients

**Figure 3:**
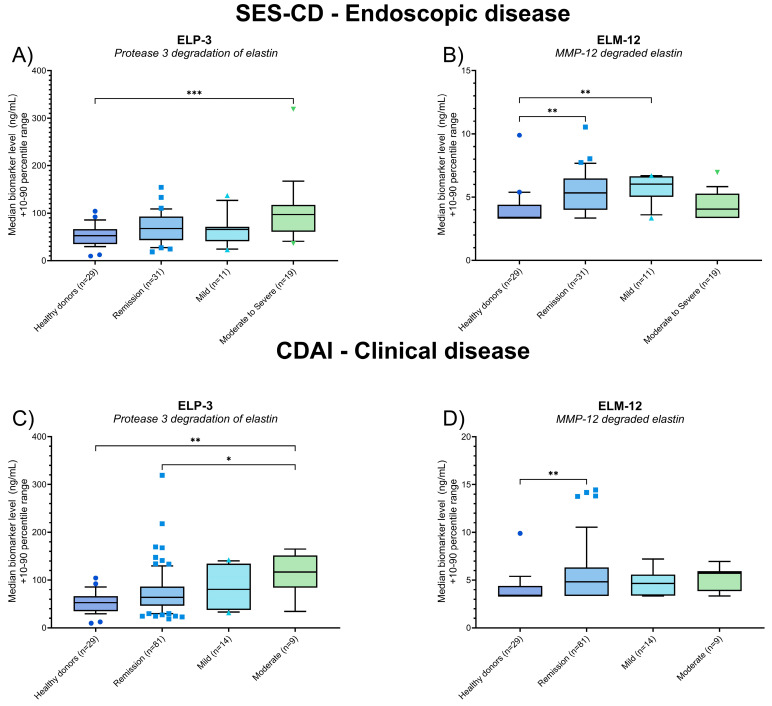
Biomarker levels of ELP-3 (**A**,**C**) and ELM-12 (**B**,**D**) quantified in the serum of healthy donors (*n* = 29) and patients with CD grouped according to their endoscopic (**A**,**B**) or clinical (**C**,**D**) disease activity. Using the SES-CD score for endoscopic disease, patients were grouped as follows: remission (*n* = 31), mild (*n* = 11), and moderate to severe (*n* = 19). Grouping was based on the CDAI score for clinical disease, with remission (*n* = 81), mild (*n* = 14), and moderate (*n* = 9). The data are plotted as box plots showcasing the median and 10th–90th percentile biomarker levels, calculating the statistical difference between the healthy donors and patients with IBD using Kruskal–Wallis one-way ANOVA and applying Dunn’s test for multiple comparisons. The statistical significance between groups is presented as * *p* < 0.05, ** *p* < 0.01, and *** *p* < 0.001.

The measured serum levels of ELP-3 and ELM-12 were plotted for the CD patients according to their endoscopic (SES-CD) and clinical disease (CDAI) scores, and patients were grouped as in remission, mild, or moderate and severe. A trend of elevated ELP-3 levels was observed in patients with moderate to severe endoscopic disease, with significantly higher levels noted compared to healthy donors (*p* = 0.0008) (Figure 3A). When plotting the serum levels of ELM-12 in CD patients according to their SES-CD scores, we observed elevated levels in patients in endoscopic remission and mild disease compared to the healthy donors (*p* = 0.001 and *p* = 0.003) (Figure 3B). Numerically elevated serum ELM-12 was observed in patients in endoscopic remission and with mild disease compared to patients with moderate to severe disease (median levels (remission, mild, moderate to severe): 5.33, 6.03, and 4.06 ng/mL) (Figure 3B). 

CD patients presenting with moderate clinical disease activity had elevated serum ELP-3 compared to patients in endoscopic remission (*p* = 0.039) and healthy donors (*p* = 0.002) (Figure 3C). Measuring serum ELM-12, a significant elevation was observed in patients in clinical remission compared to healthy donors (*p* = 0.003) (Figure 3D).

### 3.4. Biomarkers of PR3- and MMP-12-Catalyzed Elastin Degradation in Patients with UC

**Figure 4:**
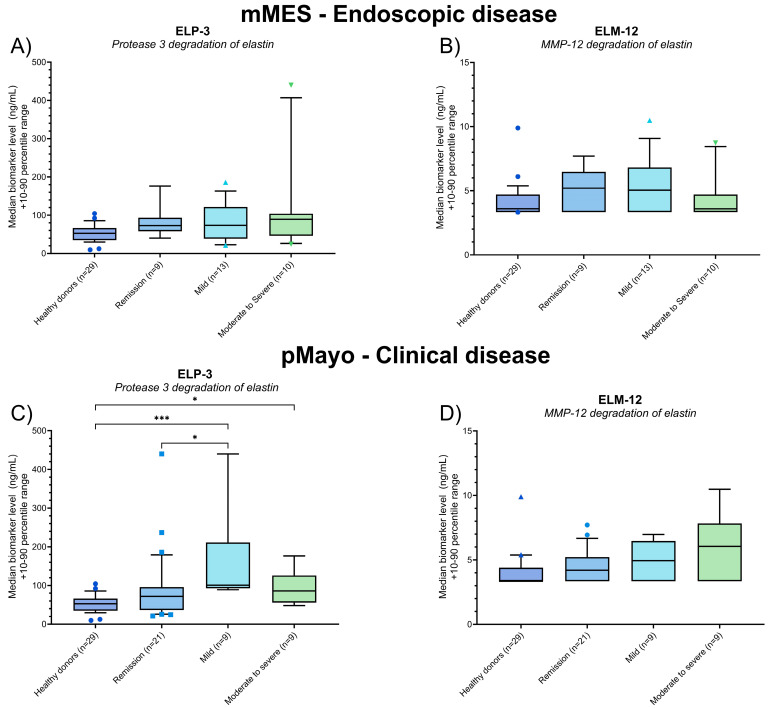
Biomarker levels of ELP-3 (**A**,**C**) and ELM-12 (**B**,**D**) quantified in the serum of healthy donors (*n* = 29) and patients with UC grouped according to their endoscopic (**A**,**B**) or clinical (**C**,**D**) disease activity. Using the mMES score for endoscopic disease, patients were grouped as follows: remission (*n* = 9), mild (*n* = 13), and moderate to severe (*n* = 10). Grouping based on the pMayo score for clinical disease was as follows: remission (*n* = 21), mild (*n* = 9), and moderate to severe (*n* = 9). The data are plotted as box plots showcasing the median and 10th–90th percentile biomarker levels, calculating the statistical difference between the healthy donors and patients with IBD using Kruskal–Wallis one-way ANOVA and applying Dunn’s test for multiple comparisons. The statistical significance between groups is presented as * *p* < 0.05 and *** *p* < 0.001.

Serum levels of ELP-3 and ELM-12 were evaluated in the UC patients according to their mMES and pMayo scores. In UC patients, ELP-3 was associated with pMayo score, demonstrating elevated levels in patients with mild disease compared to patients in clinical remission (*p* = 0.021) and healthy donors (*p* = 0.0001) (Figure 4C). Patients with moderate to severe disease presented with elevated serum ELP-3 compared to healthy donors (*p* = 0.049) (Figure 4C). No statistical difference in the serum ELP-3 or ELM-12 levels was demonstrated when assessing patients according to the mMES (ELP-3 and ELM-12) or the pMayo (ELM-12) scores (Figure 4A,B,D). 

### 3.5. Discriminating Patients with CD and UC According to Endoscopic and Clinical Disease Activity Using Elastin Degradation Biomarkers

Before evaluating the ability of the ELP-3 and ELM-12 biomarkers to correctly identify patients according to their endoscopic and clinical disease status, we performed a correlation analysis of the confounders (age, BMI, gender, smoking status, biological therapy, and ileal resection). A borderline significant and weak positive correlation between ELP-3 and the smoking status of the CD patients was demonstrated (r = 0.196, *p* = 0.047) (Supplementary Figure S1 and Table S1). Due to the overall lack of a correlation between the elastin biomarkers and confounders, we performed an ROC analysis without adjusting for the confounders. 

By performing an ROC analysis, we assessed the ability of the biomarkers to discriminate IBD patients according to their endoscopic and clinical disease scores. The patients were divided into the following groups: remission, active disease, remission and mild, and moderate and severe disease. 

**Figure 5:**
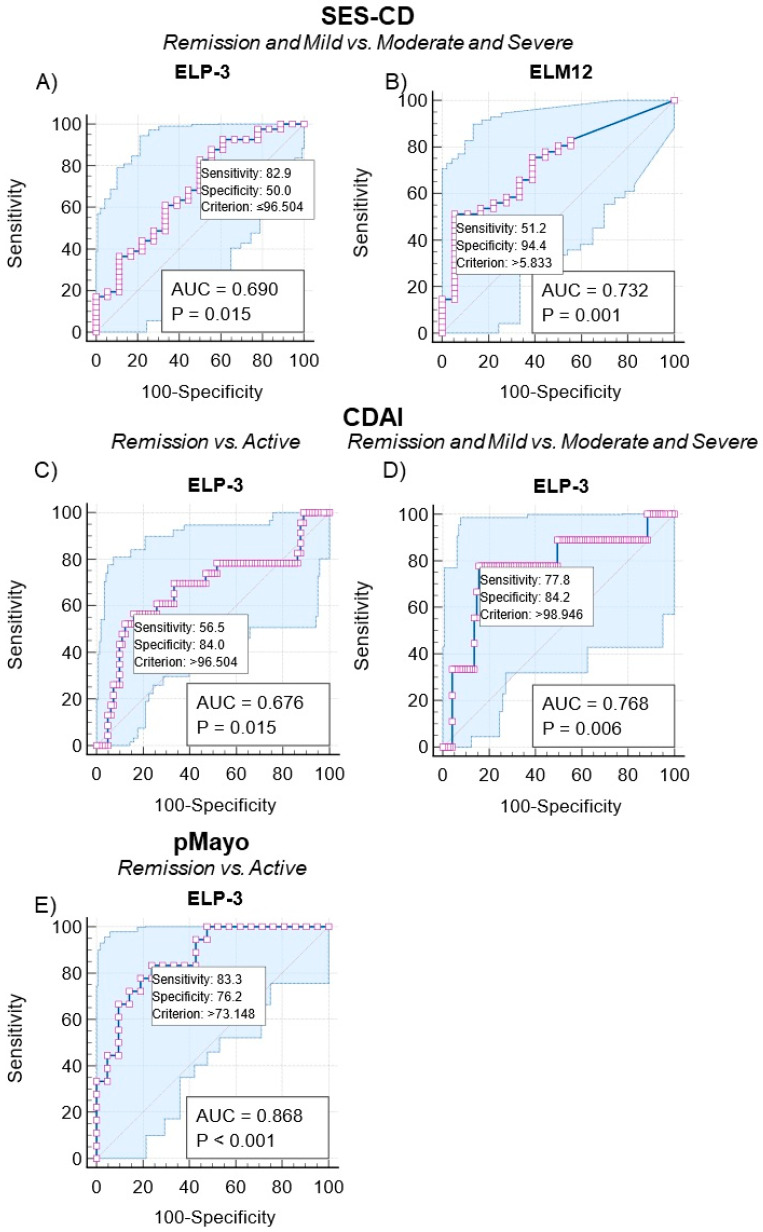
The discriminate power of the biomarkers ELP-3 and ELM-12 according to endoscopic (SES-CD) and clinical disease (CDAI and partial Mayo) activity for patients with CD and UC. The discriminate power of ELP-3 and ELM-12 was determined by receiver operating characteristics differentiating between CD patients in endoscopic remission and mild vs. moderate to severe disease (**A**,**B**), clinically active vs. remission and remission and mild vs. moderate to severe disease (**C**,**D**), and UC patients in clinical remission vs. active disease (**E**). Statistical significance was determined as *p* < 0.05.

The CD patients’ SES-CD and CDAI scores were used as markers of endoscopic and clinical disease activity. The elastin biomarkers, ELP-3 and ELM-12, provided significant discrimination when separating patients in remission and mild vs. moderate and severe endoscopic disease, with AUCs of 0.69 (sensitivity: 50.0% and specificity: 82.93%, *p* = 0.015) and 0.73 (sensitivity: 94.4% and specificity: 51.2%, *p* = 0.001) (Table 2 and Figure 5A,B). 

Using the CDAI score, ELP-3 provided significant discrimination between remission vs. active disease and remission and mild vs. moderate and severe disease. The AUC for remission vs. active disease was 0.68 (sensitivity: 56.5% and specificity: 83.9%, *p* = 0.0152), and an AUC of 0.77 (sensitivity: 77.8% and specificity: 84.2%, *p* = 0.006) was found for remission and mild vs. moderate and severe clinical disease (Table 2 and Figure 5C,D).

Utilizing the mMES score, which considers the extent of the disease, the elastin biomarkers did not provide a significant result for UC patients (Table 3). 

Measuring the ELP-3 biomarker enabled the identification of UC patients in clinical remission vs. active disease with an AUC of 0.87 (sensitivity: 83.3% and specificity: 76.2%, *p* < 0.0001). ELM-12 was unable to discern between patient groupings significantly (Table 3 and Figure 5E).

## 4. Discussion

Endoscopy remains the gold standard for monitoring mucosal healing, disease activity, therapy response, and postoperative recurrence in IBD patients. Due to the invasiveness and accompanying patient discomfort of endoscopy, frequent routine assessments are unwanted. Herein, we investigated the ability of serological biomarkers reflecting PR3 (ELP-3)- and MMP-12 (ELM-12)-catalyzed elastin degradation as surrogate markers of endoscopic and clinical disease. As endproducts of elastolytic processes, we believe these biomarkers could potentially be reflective of pathological elastin remodeling in the intestinal tissue of IBD patients. With proper validation, serological biomarkers of tissue remodeling could act as supportive tools for patient monitoring, stratification, and clinical trial enrichment and reduce the need for invasive endoscopy [21,22]. By tracing the generation of the biomarker fragments to PR3 and MMP-12, we can establish an association between the biomarkers and neutrophil and macrophage elastolytic activity.

We initially evaluated the serum levels of ELP-3 and ELM-12 in patients with CD and UC, comparing the levels to a group of healthy donors. The ELP-3 biomarker was significantly elevated in CD and UC patients, whereas ELM-12 was elevated in CD patients. The data indicate a higher degree of PR3-catalyzed elastin degradation in IBD patients and a higher degree of MMP-12-catalyzed degradation in CD patients, which was not evident in UC patients (Figure 2). While the data suggest differences in immune cell-derived protease activity and the resulting elastin degradation, the separation between IBD patients and healthy donors does not indicate a diagnostic use of ELP-3 and ELM-12. Comparing biomarker levels of the entire patient population with CD or UC negates potential differences between patients in remission and with active disease, reducing the separation of patients at the biomarker level. The lack of separation due to a heterogenous grouping was evident when we stratified patients with CD according to their SES-CD scores and CDAI scores (Figure 3) and patients with UC according to the mMES and pMayo (Figure 3) and compared biomarker levels to the healthy donors.

Subgrouping the CD patients according to endoscopic and clinical disease activity, the serum levels of ELP-3 were elevated in patients with moderate to severe disease. The ELP-3 data suggest increased neutrophil-derived PR3 activity, causing elastin degradation and potentially worsening the disease. Interestingly, the highest ELM-12 levels were observed in CD patients with endoscopic remission compared to patients with moderate to severe endoscopic disease and healthy donors (Figure 3). This indicates an association between elevated macrophage-derived MMP-12 catalyzed elastin degradation and endoscopic remission, suggesting a positive outcome for patients with high serum ELM-12. Nevertheless, studies on MMP-12 involvement in CD and intestinal inflammation demonstrate its detrimental role, which is linked to a lack of treatment response [23] and reduced histological improvement [24]. On the other hand, other research suggests that macrophages and MMP-12 may play a role in resolving tissue fibrosis, strengthening the ECM during wound healing [25,26,27], suppressing intestinal myofibroblasts, and reducing ECM deposition [28]. Hence, the ELM-12 biomarker might originate from the beneficial MMP-12-driven resolution of fibrotic ECM, including elastin.

The median levels of ELM-12 between healthy donors and CD patients with moderate to severe endoscopic disease differed by 19.4%. Despite an expected significant difference in the health status between the two groups of donors, the biomarker difference was not significant. The serum ELM-12 levels indicate low MMP-12 activity in both groups. However, the cause of a potentially low MMP-12 activity could differ significantly. Future investigations could include measurements of MMP-12 activity in healthy donors and CD patients. 

Analyzing the elastin degradation biomarkers in the UC patients, a similar trend of ELP-3 being elevated with increasing severity of clinical disease was observed (Figure 4). Active neutrophils’ detrimental effects on colonic tissue in UC patients are well-documented, and studies have shown increased levels of neutrophil-associated proteins [17,29] and antibodies targeting PR3 with endoscopic disease in UC [30], supporting ELP-3’s association with active disease. We observed no association between serum ELM-12 levels and endoscopic or clinical disease in the UC patients, as demonstrated in Figure 3. The data suggest a lower activity of MMP-12 in UC patients compared to the CD patients in this study, quantified by the ELM-12 biomarker. 

Subsequently, we evaluated the discriminative power of the biomarkers based on endoscopic and clinical disease activity using the SES-CD, CDAI, mMES, and pMayo scoring systems for CD (Table 2) and UC (Table 3). Both ELP-3 and ELM-12 provided acceptable AUCs of 0.69 and 0.73, discerning between CD patients in endoscopic remission and mild disease vs. patients with moderate to severe disease. The Youden index score demonstrates the opposite association with the two biomarkers, as indicated in Figure 3, Table 3, and Figure 5A,B. The Youden index biomarker cut-off value for ELP-3 was <96.5 ng/mL and ≥5.83 ng/mL for ELM-12. Thus, remission and mild disease patients would be identified by reduced PR3 activity and elevated MMP-12 activity, quantified by the neoepitope-specific biomarkers ELP-3 and ELM-12. Similarly, we observed an acceptable ability to discriminate CD patients in remission and with mild clinical activity from patients with moderate to severe disease using serum ELP-3 levels. The data substantiate ELP-3 as a potential biomarker of active disease in CD related to neutrophil-catalyzed elastin degradation. 

Discriminating the UC patients, ELP-3 provided an AUC of 0.87, distinguishing remission vs. active disease based on the pMayo score (Table 3 and Figure 5E). No association with endoscopic disease was determined in the current study, and an association with the pMayo score indicates a strong relation to the clinical part rather than endoscopic findings. The elevation of ELP-3 may be associated with rectal bleeding, a component of the pMayo score assessment, which could reflect proteolytic elastin degradation driven by neutrophils and PR3.

Since endoscopy is not routinely performed at every visit, clinical scorings such as the pMayo can provide noninvasively obtained information on the patient’s disease status, showing a good correlation to the Full Mayo score [31] and the relationship between rectal bleeding and endoscopic findings [32]. ELP-3 may complement the current clinical score by adding a functional measure of neutrophil- and PR3-facilitated elastin degradation. However, our study did not dissect the pMayo score to determine whether ELP-3 was specifically related to rectal bleeding or stool frequency. Additional research is needed to understand the association between ELP-3 and endoscopic disease in UC.

Our data point towards the potential of ELP-3 and ELM-12 as surrogate markers for identifying CD patients in endoscopic remission, potentially reducing the need for routine endoscopy. Moreover, ELP-3 may be applied for UC patients identifying patients with an active disease. However, the biomarkers in the current study are outperformed by the collagen remodeling biomarkers evaluated in the same study by Domislovic et al. [12]. Although the collagen biomarkers in the current study outperform the elastin degradation biomarker, future studies could attempt a combination of the biomarkers. Whereas the biomarkers investigated by Domislovic et al. reflect collagen remodeling, ELP-3 and ELM-12 can provide insights into neutrophil- and macrophage-catalyzed elastin degradation. 

Our study has several limitations and requires further investigation. Evaluating the association of elevated ELM-12 with endoscopic remission is necessary, as research on MMP-12’s proteolytic activity in CD demonstrates its link to active disease. Moreover, the study population included patients from a tertiary center with advanced CD, where a significant proportion was on biologics and had severe complications. As a result, it may not fully represent the general CD population, limiting the generalizability of the findings. Additionally, our analysis’s small number of UC patients reduces its statistical power.

While ELP-3 showed acceptable AUCs in identifying UC patients with clinically active disease, the limited patient numbers affected the precision of our analysis. The availability of endoscopic data for CD and UC patients was advantageous, allowing evaluation with the mMES score. However, the limited endoscopic data hindered a comprehensive biomarker evaluation.

Mucosal healing is a crucial treatment goal in IBD, and serological biomarkers such as ELM-12, ELP-3, and collagen remodeling biomarkers hold potential as surrogate markers. Their direct association with tissue turnover driven by specific cells and proteases involved in ECM protein remodeling connects them to mucosal and even transmural healing. Combining these biomarkers with other serological markers could lead to optimized models as supportive tools in clinical studies [12,13,14,22,33,34].

Our investigation reveals an association between the serological biomarkers of elastin degradation, ELM-12 generated by MMP-12, and ELP-3 generated by PR3 and endoscopic disease in CD and clinical disease in UC patients. 

## Figures and Tables

**Table 1:** Patient demographics for patients with CD and UC and healthy donors.

Variable	CD (*n* = 104)	UC (*n* = 39)	Healthy Donors (*n* = 29)	*p*-Values
Age, years (IQR)	36 (17.75)	38 (24)	39.5 (14)	*p* > 0.999
Gender, male, *n* (%)	66 (63.5)	23 (59)	17 (58.6)	*p* = 0.829
BMI, kg/m^2^ (IQR)	22.9 (6.39)	23.7 (6.3)		*p* = 0.934
Smoking, yes, *n* (%)	23 (22.1)	5 (12.8)		*p* = 0.246
Montreal L (CD), *n* (%) L1/L2/L3	17 (17.5)/17 (17.5)/63 (65.0)	/		
Montreal B (CD), *n* (%) B1/B2/B3	34 (39.5)/35 (40.7)/17 (19.8)	/		
Montreal E (UC), *n* (%) E1/E2/E3	/	4 (10.0)/12 (30.0)/24 (60.0)		
Endoscopic activity (SES-CD and MES), *n* (%) Remission/mild/moderate to severe	31 (50.8)/11 (18.0)/19 (31.1)	6 (18.8)/9 (28.1)/17 (53.1)		
Modified Mayo Endoscopic Score, *n* (%) Remission/mild/moderate to severe	/	9 (28.1)/13 (40.6)/10 (31.3)		
Clinical activity (CDAI and pMayo), *n* (%) Remission/mild/moderate to severe	81 (77.9)/14 (13.5)/9 (8.7)	21 (53.8)/9 (23.1)/9 (23.1)		
Biological therapy, yes, *n* (%)	62 (59.6)	27 (56.3)		*p* = 0.336
Ileal resection, yes, *n* (%)	52 (50.0)	/		
Disease duration (years) (IQR)	9.0 (12.0)	6.0 (7.0)		*p* = 0.014
CRP, mg/L (IQR)	2.5 (5.1)	2.6 (7.8)		*p* = 0.884

Abbreviations: BMI, body mass index; CD, Crohn’s disease; CDAI, Crohn’s Disease Activity Index; CRP, C-reactive protein; UC, ulcerative colitis; IQR, interquartile range; MES, Mayo Endoscopic Score; pMayo, partial Mayo score; SES-CD, Simple Endoscopic Score for Crohn’s Disease.

**Table 2:** An overview of the discriminative power of the elastin degradation biomarkers by receiver operating characteristic analysis between patients with Crohn’s disease categorized based on their SES-CD and CDAI scores for endoscopic and clinical scoring.

Disease Scoring	Patient Grouping	Biomarker	AUC	AUC CI	Sensitivity (95% CI)	Specificity (95% CI)	Youden Index	*p*-Value
SES-CD	Remission vs. active	ELP-3	0.59	0.45 to 0.71	93.5 (78.6–99.2)	28.6 (13.2–48.7)	<110.27	*p* = 0.260
		ELM-12	0.61	0.47 to 0.73	38.7 (21.8–57.8)	85.7 (67.3–96.0)	≥6.29	*p* = 0.141
	Remission and mild vs. moderate and severe	ELP-3	0.69	0.56 to 0.80	82.9 (67.9–92.8)	50.0 (26.0–74.0)	<96.50	*p* = 0.015
		ELM-12	0.73	0.60 to 0.84	51.2 (35.1–67.1)	94.4 (72.7–99.9)	≥5.83	*p* = 0.001
CDAI	Remission vs. active	ELP-3	0.68	0.58 to 0.76	83.9 (74.1–91.2)	56.5 (34.5–76.8)	<96.50	*p* = 0.015
		ELM-12	0.52	0.42 to 0.62	35.80 (25.4–47.2)	82.6 (61.2–95.0)	≥5.83	*p* = 0.801
	Remission and mild vs. moderate and severe	ELP-3	0.77	0.68 to 0.85	84.2 (75.3–90.9)	77.8 (40.0–97.2)	<98.95	*p* = 0.006
		ELM-12	0.54	0.44 to 0.64	67.4 (57.0–76.6)	55.6 (21.2 86.3)	≥5.70	*p* = 0.689

Abbreviations: AUC, area under the curve; CDAI, Crohn’s Disease Activity Index; CI, confidence interval; SES-CD, Simple Endoscopic Score Crohn’s disease.

**Table 3:** An overview of the discriminative power of the elastin degradation by receiver operating characteristic analysis between patients with ulcerative colitis categorized by their mMES and pMayo scores for endoscopic and clinical disease.

Disease Scoring	Patient Grouping	Biomarker	AUC	AUC CI	Sensitivity (95% CI)	Specificity (95% CI)	Youden Index	*p*-Value
mMES	Remission vs. active	ELP-3	0.52	0.33 to 0.70	11.1 (0.3–48.2)	60.9 (38.5–80.3)	<54.88	*p* = 0.875
		ELM-12	0.57	0.38 to 0.74	66.7 (29.9–92.5)	60.9 (38.5–80.3)	≥4.32	*p* = 0.539
	Remission and mild vs. moderate and severe	ELP-3	0.52	0.34 to 0.70	59.1 (36.4–79.3)	60.0 (26.2–87.8)	<82.91	*p* = 0.846
		ELM-12	0.62	0.43 to 0.78	59.1 (36.4–79.3)	80.0 (44.4–97.5)	≥4.32	*p* = 0.261
pMayo	Remission vs. active	ELP-3	0.87	0.72 to 0.96	76.2 (52.8–91.8)	83.3 (58.6–96.4)	<73.15	*p* < 0.0001
		ELM-12	0.65	0.48 to 0.79	90.5 (69.6–98.8)	50.0 (26.0–74.0)	<5.59	*p* = 0.107
	Remission and mild vs. moderate and severe	ELP-3	0.65	0.48 to 0.79	36.7 (19.9–56.1)	100.0 (66.4–100.0)	<40.97	*p* = 0.139
		ELM-12	0.68	0.51 to 0.82	83.3 (65.3–94.4)	66.7 (29.9–92.5)	<5.59	*p* = 0.142

Abbreviations: AUC, area under the curve; CI, confidence interval; mMES, modified Mayo Endoscopic Score; pMayo, partial Mayo.

## Data Availability

The data underlying this article cannot be shared publicly due to the privacy of individuals that participated in the study. The data can be shared upon reasonable request to the corresponding author.

## Supplementary Materials

The following supporting information can be downloaded at: https://www.mdpi.com/article/10.3390/jcm13010021/s1, Figure S1: Correlation of ELP-3 (A and C) and ELM-12 (B and D) levels and confounders of patients with CD (*n* = 104) and UC (*n* = 39). We performed a nonparametric Spearman correlation analysis to investigate the correlation of the biomarkers, ELP-3 and ELM-12, with the demographic data of the patient’s age, body mass index, gender, biological therapy, smoking status, ileal resection, and disease duration (years); Table S1: Provides an overview of the correlations between the biomarkers, ELP-3 and ELM-12 and the confounders: age, body mass index, gender (male/female, biological therapy (yes/no), smoking status (no, yes, ex), ileal resection (no/yes), and disease duration (years).
