# Supplementary material for: Blood-Based Biomarkers Reflecting Protease 3 and MMP-12 Catalyzed Elastin Degradation as Potential Noninvasive Surrogate Markers of Endoscopic and Clinical Disease in Inflammatory Bowel Disease
Source: J Clin Med. 2023 Dec 19;13(1):21. doi: 10.3390/jcm13010021 (PMC10779348; doi:10.3390/jcm13010021)
Supplement: Supplementary file 1 [file jcm-13-00021-s001.zip › jcm-2745936-supplementary.pdf]

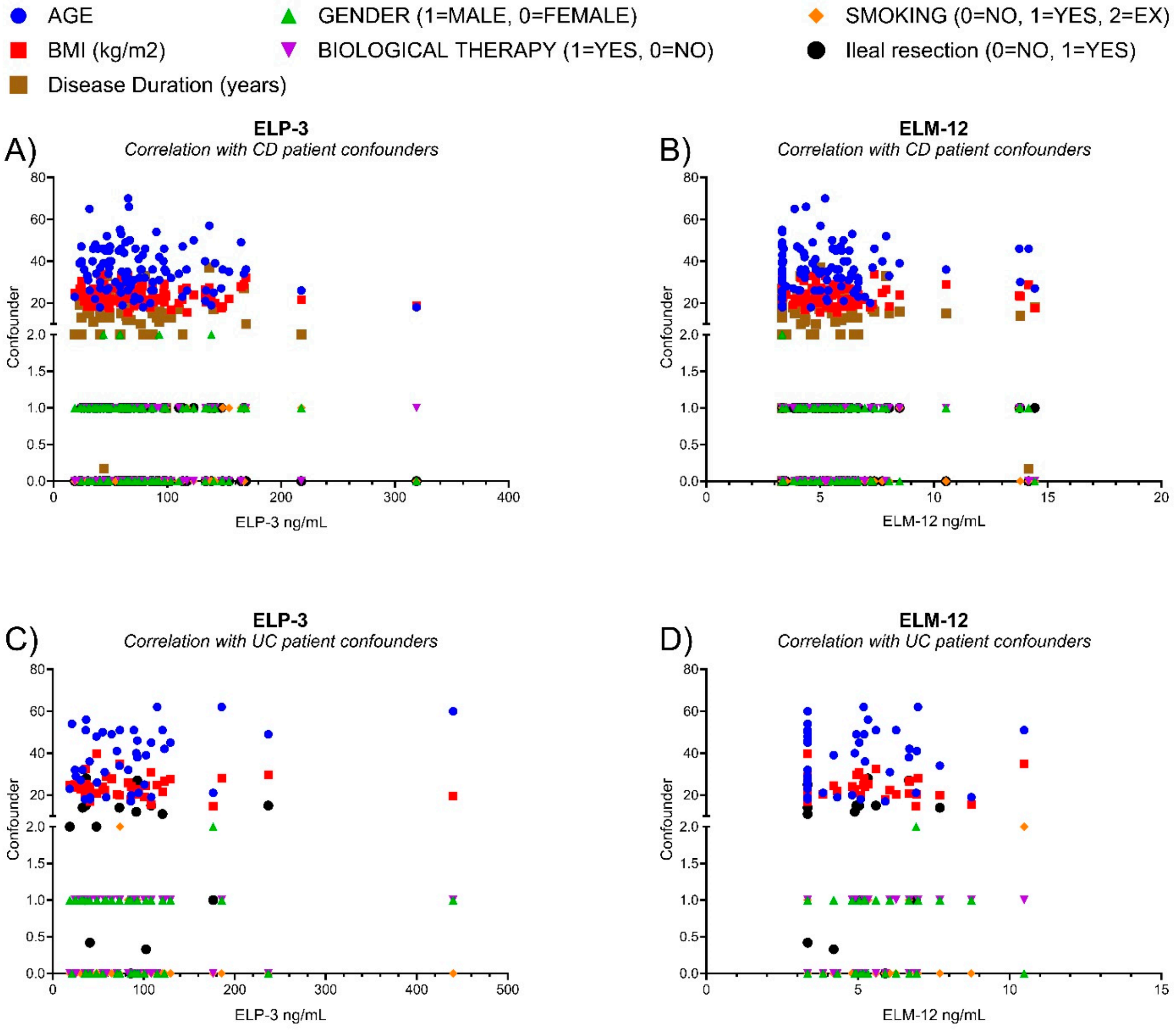

Figure S1. Correlation of ELP-3 (A and C) and ELM-12 (B and D) levels and confounders of patients with CD (n=104) and UC (n=39). We performed a nonparametric Spearman correlation analysis to investigate the correlation of the biomarkers, ELP-3 and ELM-12, with the demographic data of the patient's age, body mass index, gender, biological therapy, smoking status, ileal resection, and disease duration (years).

Table S1: Provides an overview of the correlations between the biomarkers, ELP-3 and ELM-12 and the confounders: age, body mass index, gender (male/female, biological therapy (yes/no), smoking status (no, yes, ex), ileal resection (no/yes), and disease duration (years).

Crohn's Disease				
**Biomarker**	**Confounder**	**r**	**95% CI**	**p-value**
**ELP-3**	Age	-0.15	-0.34 to 0.05	P = 0.130
	Body mass index	-0.07	-0.26 to 0.13	P = 0.497
	Gender	-0.06	-0.26 to 0.15	P = 0.574
	Biological therapy	-0.11	-0.30 to 0.09	P = 0.268
	Smoking status	0.20	-0.003 to 0.38	P = 0.047
	Ileal resection	-0.19	-0.37 to 0.010	P = 0.055

	Disease duration	-0.11	-0.30 to 0.09	P = 0.271
**ELM-12**	Age	0.04	-0.16 to 0.24	P = 0.696
	Body mass index	-0.05	-0.24 to 0.15	P = 0.637
	Gender	-0.12	-0.31 to 0.084	P = 0.232
	Biological therapy	0.013	-0.19 to 0.21	P = 0.899
	Smoking status	0.12	-0.08 to 0.31	P = 0.235
	Ileal resection	0.12	-0.08 to 0.31	P = 0.213
	Disease duration	0.02	-0.18 to 0.22	P = 0.868
**Ulcerative Colitis**				
**ELP-3**	Age	0.26	-0.06 to 0.53	P = 0.100
	Body mass index	-0.08	-0.39 to 0.24	P = 0.609
	Gender	0.06	-0.26 to 0.37	P = 0.705
	Biological therapy	0.09	-0.24 to 0.39	P = 0.590
	Smoking status	0.01	-0.31 to 0.33	P = 0.939
	Disease duration	-0.00	-0.32 to 0.31	P = 0.974
**ELM-12**	Age	0.12	-0.21 to 0.42	P = 0.468
	Body mass index	-0.07	-0.38 to 0.25	P = 0.664
	Gender	-0.16	-0.45 to 0.17	P = 0.332
	Biological therapy	0.03	-0.29 to 0.34	P = 0.873
	Smoking status	0.12	-0.20 to 0.42	P = 0.439
	Disease duration	-0.09	-0.39 to 0.24	P = 0.590
